# Emotion Recognition from Physiological Signals Collected with a Wrist Device and Emotional Recall

**DOI:** 10.3390/bioengineering10111308

**Published:** 2023-11-11

**Authors:** Enni Mattern, Roxanne R. Jackson, Roya Doshmanziari, Marieke Dewitte, Damiano Varagnolo, Steffi Knorn

**Affiliations:** 1 Chair of Control Engineering, Technische Universität Berlin, Straße des 17. Juni 135, 10623 Berlin, Germany; enni.mattern@tu-berlin.de (E.M.); jackson@tu-berlin.de (R.R.J.); knorn@tu-berlin.de (S.K.); 2 Department of Engineering Cybernetics, Norwegian University of Science and Technology (NTNU), Høgskoleringen 1, 7034 Trondheim, Norway; roya.doshmanziari@ntnu.no; 3 Department of Clinical Psychological Science, Maastricht University, Minderbroedersberg 4–6, 6211 LK Maastricht, The Netherlands; marieke.dewitte@maastrichtuniversity.nl; 4 Department of Information Engineering, University of Padova, Via VIII Febbraio, 2, 35122 Padova, PD, Italy

**Keywords:** emotion recognition, physiological signals, machine learning, support vector machines, medical wristband

## Abstract

Implementing affective engineering in real-life applications requires the ability to effectively recognize emotions using physiological measurements. Despite being a widely researched topic, there seems to be a lack of systems that translate results from data collected in a laboratory setting to higher technology readiness levels. In this paper, we delve into the feasibility of emotion recognition beyond controlled laboratory environments. For this reason, we create a minimally-invasive experimental setup by combining emotional recall via autobiographical emotion memory tasks with a user-friendly Empatica wristband measuring blood volume pressure, electrodermal activity, skin temperature, and acceleration. We employ standard practices of feature-based supervised learning and specifically use support vector machines to explore subject dependency through various segmentation methods. We collected data from 45 participants. After preprocessing, using a data set of 134 segments from 40 participants, the accuracy of the classifier after 10-fold cross-validation was barely better than random guessing (36% for four emotions). However, when extracting multiple segments from each emotion task per participant using 10-fold cross-validation (i.e., including subject-dependent data in the training set), the classification rate increased to up to 75% for four emotions but was still as low as 32% for leave-one-subject-out cross-validation (i.e., subject-independent training). We conclude that highly subject-dependent issues might pose emotion recognition.

## 1. Introduction

In many treatments for medical and psychosomatic disorders, such as physical therapy and other relaxation techniques, there is often a lack of consistent feedback on how well a patient responds to the therapy. This lack of feedback often leads to high drop-out rates and irregular training, which hinders the therapy’s ability to constantly yield an improved physical or psychological state. Moreover, therapies for which a patient’s psycho-physiological state is of particular importance require a human-centered design approach to effectively address subjective pain and emotions experienced by patients. For example, physiotherapy using vaginal dilators to treat genital pain and penetration disorders or re-validation treatment after genital surgery are invasive and may cause high levels of discomfort [1]. There is hence, an evident need for emotional feedback.

The concept of affective engineering extends beyond the scope of the vaginal dilation therapy example above and encompasses various domains where understanding and incorporating human emotions are crucial. For example, Balters and Steinert [2] highlight the need for novel methods that account for the influence of human emotions, which often lead to irrational behavior. They propose that physiological signals and machine learning (ML) techniques could be fundamental in advancing research in this field, as these signals cannot be intentionally influenced by the subject. The underlying assumption is that our body functions are regulated by the autonomic nervous system, which is in turn influenced by emotions [3]. Thus, by measuring physiological signals, emotional states can be inferred. Consequently, the emergence of user-friendly devices capable of non-invasive measurements presents an excellent opportunity to incorporate biofeedback and emotion reactivity into human-machine interactions and to personalize medical therapies. However, the integration of using such information as a form of feedback or to impact the control of a smart device is currently lacking. Further, there is a clear incentive to integrate the collection and classification of physiological signals in home-based physical therapy sessions. Consequently, it is of high importance to use minimally invasive and user-friendly data collection methods.

### 1.1. State of the Art

Emotion recognition using physiological signals is an extensively researched area. Using various approaches, both binary [4,5,6,7,8,9,10] and multi-class classification [11,12,13] have achieved high accuracy rates exceeding 80%. Nevertheless, cross-study results comparison poses a challenge due to varying factors such as the number of subjects, subject-dependent results, the chosen machine learning and validation methods, the signals used, emotion elicitation techniques, and emotion models. For a comprehensive review covering these aspects, please see [14,15]. For this study, it is particularly important to us that the data collection method is applicable outside of a laboratory setting. The sensors and the methods for emotion elicitation play a vital role in the technological readiness level of the system. Hence, we review these categories with a focus on their applicability to a minimally invasive scenario.

#### 1.1.1. Signals

The autonomic nervous system (ANS) regulates “somatic homeostasis and regulates visceral activity, such as the heart rate, digestion, respiratory rate, salivation, perspiration, pupil diameter, micturition, and sexual arousal” [2]. Hence, it is often assumed that measuring these physiological reactions can offer insights into emotional states, and there have even been attempts to establish fixed relationships between certain signals or features and emotional changes [15]. It is important to note, however, that not all of the mediated reactions indicate emotional changes [3], and we must acknowledge that any such correlations are likely to be highly nonlinear.

Due to the complex relationship between emotions and body responses, most studies utilize multiple physiological signals. As per a review conducted by Bota et al. [14], the most frequently used sensor systems and signals (listed from the most to the least often used) include electrodermal activity (EDA), electrocardiography (ECG), respiration rate (RESP), electroencephalography (EEG), electromygraphy (EMG), skin temperature (SKT), acceleration (ACC), blood volume pulse (BVP), and electrooculography (EOG). However, it is crucial to note that many of these signals are only viable in a laboratory setting, e.g., due to electrode placement challenges and the need for specialized equipment which often reduces the mobility of the participants. In settings that are closer to a real-life scenario, the emphasis shifts from, e.g., EEG features to cardiovascular and EDA features.

With this objective in mind, photoplethysmography (PPG) sensors, measuring BVP, provide essential heart rate data. These non-invasive sensors, found in smartwatches and medical wearables, aren’t widely used in emotion recognition due to their sensitivity to movement. Nonetheless, PPG sensors have been implemented in several studies [4,12], and sometimes in conjunction with other non-invasive sensors for EDA [11,16]. One of the most widely used open databases incorporating PPG measurements is the database for emotion analysis using physiological signals (DEAP) [17], as shown in studies such as [5,9,10,18]. Yet, data transferability to other studies using a BVP signal remains challenging due to DEAP’s use of a fingertip PPG sensor and the inclusion of other measures like ECG or EEG for classification.

There are instances where researchers conducted short-term emotion recognition with a minimally invasive setup, for example, [5] used only a 10 s BVP signal and achieved accuracy rates exceeding 80% for binary classification. Moreover, ppg sensors embedded in wearables have been applied in real-life situations by [7,18,19]. Both [7,18] utilized a measurement wristband developed by Empatica. While these devices offer the advantage of portability and easy setup, participants often need to remain still to get a clean PPG signal [20] and researchers are required to deal with noisy signals, e.g., by disregarding corrupted parts [7].

#### 1.1.2. Emotion Models and Elicitation

Emotions are typically defined by two types of models: discrete emotion spaces and continuous dimensions [14]. Discrete models assume emotions can be grouped into categories like “sad” or “happy”, but this can lead to individual interpretation differences due to factors such as cultural backgrounds [14]. Continuous models, on the other hand, measure emotions along one or more axes, which simplifies emotion comparison [14]. According to Bota et al. [14], the most popular model that uses continuous dimensions is the valence-arousal model by Russell [21], where valence represents the positivity or negativity of an emotion, and arousal indicates its intensity.

The majority of emotion elicitation methods occur in a laboratory setting, utilizing various stimuli such as pictures, films, sounds, words, or recall schemes [14,22]. Just as with the emotion models, the environment of the participants, including factors like language and cultural background, will considerably impact the emotion elicitation process inherent to the stimuli of some of these methods. Though recall schemes, such as the autobigraphical emotion memory task (AEMT), offer an established way for emotion elicitation without relying on external stimuli [23], they are notably underrepresented in emotion recognition studies. As highlighted by Bota et al. [14], a mere two out of over seventy reviewed studies employed any form of recall scheme. For instance, Picard et al. [13] effectively employed a recall scheme over two decades ago, achieving correct rates of 81% across eight emotion classes for a single subject. Yet, these results have been infrequently reproduced. Chanel et al. [24] worked with a group of 10 participants and achieved a correct recognition rate of up to 50% for four emotions using a subject-dependent model.

#### 1.1.3. Methods for Classification and Validation

In emotion recognition using physiological signals, methods generally fall into two categories: traditional machine learning and deep learning [14]. Traditional machine learning involves signal preprocessing, feature engineering, feature fusion, feature-dependent classification, and validation. The aim of signal preprocessing is to reduce noise while retaining relevant information. Feature engineering, the subsequent step, is intended to maximize the informative content of the preprocessed signals [14]. After feature computation, dimensionality reduction methods are typically applied to avoid the curse of dimensionality. Selecting appropriate features and dimensionality reduction techniques is critical, and the classifier’s success heavily depends on these choices. Features are usually fused into a single vector, which is then used to train and validate the classifier. Most commonly, support vector machine (SVM) algorithms are employed as a supervised learning (SL) method [14], as demonstrated in [6,7,8,10,11,12,24].

In contrast to feature-dependent classifiers, deep learning (DL) methods can perform pattern recognition directly on preprocessed signals without feature engineering [18]. For instance, convolutional neural networks (CNNs) can be used to learn feature-like representations [5,25]. Additional representation learning algorithms like autoencoders, utilized in [16], serve as a method for dimensionality reduction, where the autoencoder’s output feeds into the CNN. While such techniques can greatly reduce the time-consuming steps of preprocessing and feature engineering, DL methods have the limitation of behaving like a black box and require large data sets for training [14], which are often unavailable in medical experiments.

#### 1.1.4. Validation, Segmentation, and Subject-Dependency

After the classifier is trained and hyperparameters are learned, the model is typically validated on a test data set to measure performance on unseen data [14]. Common cross validation (CV) methods include *k*-fold, leave-one-subject-out (LOSO), and leave-one-out (LOO) CV. In the development of patient models, since we are using multiple experiments from the same subject, the CV method is of particular importance. Notably, both *k*-fold and LOO CV methods include subject data in the validation set. Thus, it is important to note that to be able to claim subject-independent results, LOSO CV should be used, where the classifier is tested on data exclusively from one subject, with none of this subject’s data used in training [14]. The subject-dependency in the context of *k*-fold cross-validation becomes increasingly pronounced when multiple trials are conducted per emotion or class, and even more so when several samples are derived from a single trial. In this context, the use of LOSO CV for subject-independent results is absolutely crucial.

Table 1 provides an overview of some studies, including their segmentation approaches, validation methods, trained models, and achieved accuracy rates. As mentioned previously, conducting a fair cross-study comparison is a complex issue. Nevertheless, we anticipate that this table can give a qualitative perspective on the significance of segmentation and validation methodologies. The segment number indicates the total count of segments/data points utilized for training and testing and can be inferred from other numbers. For instance, the number of subjects and labels used for emotion elicitation (which could refer to various emotions or high/low arousal levels) can be an indicator. Furthermore, some researchers conducted multiple trials per label, such as presenting several movie clips to elicit an emotion. In some cases, researchers split a single continuous segment from one trial into multiple segments for data analysis. However, the number of studies listed in the table is limited as not all researchers detail their segmentation methods. It becomes apparent that, despite the overall aim to achieve subject-independent results for greater generalizability, only a few studies utilize LOSO.

### 1.2. Proposed Approach and Novelty

As discussed above, numerous emotion recognition approaches have been tested, but only a few of them employ measuring devices that can be easily applied outside laboratory settings. Furthermore, to the best of our knowledge, there is a scarcity of experimental evidence that is collected through setups that accurately replicate real-life scenarios and that stimulate the persons by evoking self-triggered emotions. One of our contributions is thus addressing this lack by proposing a novel methodology for the design of experiments that combines emotional recalls with non-invasive wrist measurement devices. This approach is designed so to closely approximate real-life scenarios while ensuring minimal intrusion for medical therapies.

Another contribution is examining which potential factors may be the root of the lack of emotion recognition systems with high technology readiness levels, and applicable to non-laboratory settings. Most interestingly, we have seen that while most researchers work with data sets from multiple subjects, only a few researchers use LOSO CV. Moreover, we see that there is a lack of studies that clearly present the differences (e.g., by presenting the results of a leave-one-subject-out validation along with the results of *k*-fold) between subject-independent and subject-dependent evaluation and critically discuss their results. Our analyses show though that LOSO CV is needed to obtain subject-independent results. Hence, our analyses eventually check the limitations of existing approaches in emotion recognition by examining the impact of subject dependency by testing multiple data sets using different methods for segmentation and validation.

### 1.3. Structure of the Manuscript

We continue the manuscript by outlining the minimally invasive data collection process we adopted, followed by the signal preprocessing and feature selection methods. We then briefly discuss the classification and validation methods. In Section 3, we provide a comprehensive comparison of different data sets, feature sets, and validation methods. We compare and discuss our results compared to the standing literature, with a particular emphasis on segmentation and validation methods. Finally, we provide an outlook for emotion recognition experiments and the methods adopted in this area of research, with a specific focus on the efficacy of methods for producing subject-independent results.

## 2. Materials and Methods

**Figure 1:**
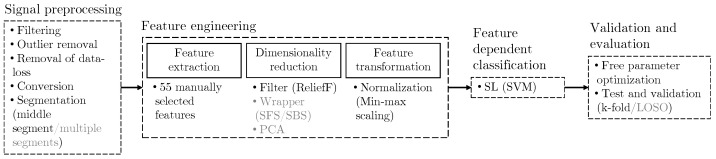
Overview of methods for signal processing, feature engineering, classification, and validation used in this study. The subject-independent approach is highlighted in black, while methods used for the purpose of validation and investigation of subject-dependency are depicted in grey.

Given our emphasis on improving experimental design and investigating subject-dependency in emotion recognition, our strategy is to use machine learning methods already proven effective in prior studies. Recognizing that recall methods are time-consuming during experiments, which consequently restricts the collection of a large data set, our study employs traditional machine learning methods, i.e., SVM. This algorithm has been frequently and successfully employed in past research. The overall structure of the methods used in this study is depicted in Figure 1. The steps for our approach to get a subject-independent method, which we favor due to the generalizability of the approach, are represented in black. Importantly, we choose to create a data set that only extracts a single segment per trial. The effect of this is that, since we only ran one trial per emotion, it is possible to use *k*-fold validation for the purpose of checking the stability of subject-independent models. In other words, the adopted strategy enables the formation of training sets for which there are no multiple segments with the same emotion from the same participant.

Besides validating our methodology, we recall that one of our goals is to investigate why there seems to be a lack of high technology readiness levels of emotion detectors in commerce. To perform this investigation we used the alternative strategies illustrated in grey in Figure 1. Most importantly, we wanted to examine the significance of subject dependency in the final classification results. For the subject-dependent approach, we extracted multiple segments per trial and tested these data sets using *k*-fold, and LOSO for comparison. We also experimented with different techniques for dimensionality reduction, since this key aspect of our machine learning process could have a significant impact on the performance of the classifier.

### 2.1. Experimental Protocol

**Figure 2:**
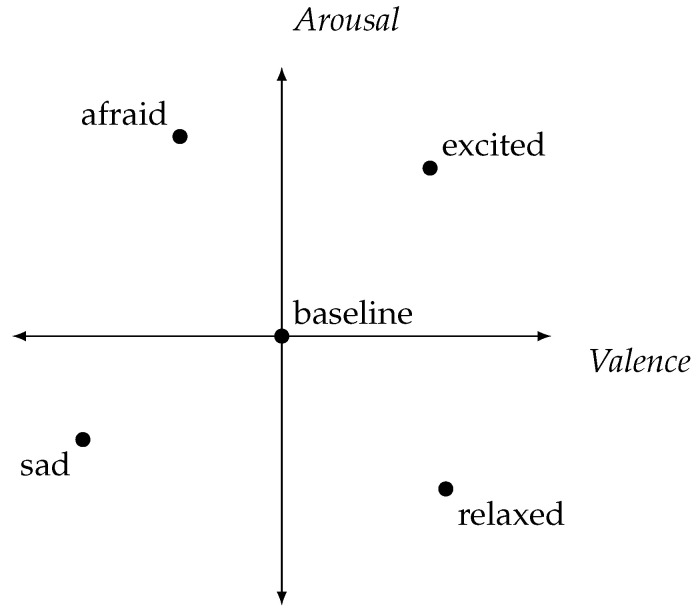
Overview of the chosen elicited emotions in the valance-arousal planes according to Russell [21].

In our pursuit of a minimally invasive setup, we employed the Empatica E4 [28], a device that captures non-invasive metrics including BVP, EDA, SKT, and ACC. Our emotion elicitation technique was inspired by the AEMT, wherein participants recall and write about personal experiences to generate specific emotions. For our experiment, we sought to elicit four distinct emotions: sadness, excitement, fear, and relaxation, along with a baseline measurement. As shown in Figure 2, the chosen emotions are distant and lie in different quadrants of the valance-arousal plane. The emotion theory developed by Russell [21] suggests that similar emotions will still fall within the same quadrant, allowing for good separability even in instances where participants may experience a mix of emotions.

**Figure 3:**
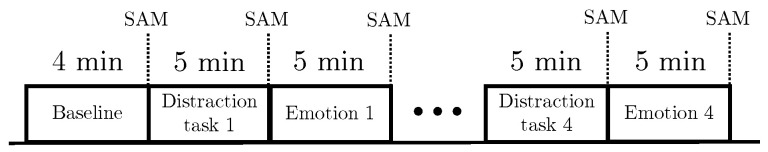
Experimental protocol for a random order of emotion excitation tasks performed for five minutes each and intermittent distraction tasks for five minutes.

The experimental protocol, as illustrated in Figure 3, starts with the participant writing about neutral topics, such as their morning routine or personal facts, to establish a baseline that corresponds to a neutral state on the valence-arousal plane. The participant then completes a randomized distraction task (chosen from options such as Sudoku, a labyrinth, finding all ‘A’s in a text, or copying geometric shapes from one sheet to another), followed by their first randomized emotion task. This sequence was repeated until all four emotions were elicited. After each distraction and emotion task, participants rated their level of valence and arousal using the picture-based self-assessment manikin (SAM) [29]. Due to the sensitivity of the PPG sensor to wrist movement, participants were instructed to keep their wrists still during the experiment. Following this protocol, we collected data from 45 participants (7 female, 19 male, 19 not answered, ranging in age from 12 to 77, with an average age of 29.7 years).

### 2.2. Preprocessing

**Figure 4:**
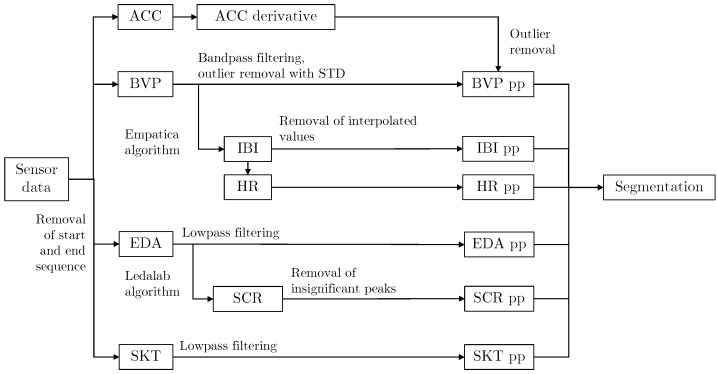
Overview of preprocessing for all signals. The preprocessed signals are denoted with a “pp” after the name. The next step after signal processing is segmentation.

Figure 4 illustrates the various preprocessing methods we applied to the different signals. Due to the structure of the experiment, see Figure 3, the participants needed to interact with a computer to answer the SAM assessment and change between distraction and emotional tasks. To eliminate the potential outliers in the data caused by the unavoidable movement, we removed the non-relevant start and end sequences from each signal before the preprocessing stage.

For the BVP signal, we used a 4th-order Butterworth bandpass filter with cutoff frequencies of 1 Hz and 8 Hz to minimize both high-frequency measurement noise and low-frequency movement noise, following the method used by Domínguez-Jiménez et al. [11]. Then, we eliminated outliers by leveraging the ACC information. Movement-related points were classified by computing the jerk and identifying any absolute values above 2 m s^−3^. All BVP signal values within a 200-point window after any derivative of the ACC exceeded this jerk threshold were tagged as corrupted. This 200-point window corresponds to roughly 3 s given a sample rate of 64 Hz. Another outlier removal step addressed outliers not due to wrist movements by calculating the standard deviation and marking data points outside of a threshold of three times the standard deviation. The inter-beat interval (IBI) and heart rate (HR) were computed from the BVP signal using Empatica’s proprietary algorithm, with interpolated IBI signal values being removed.

**Figure 5:**
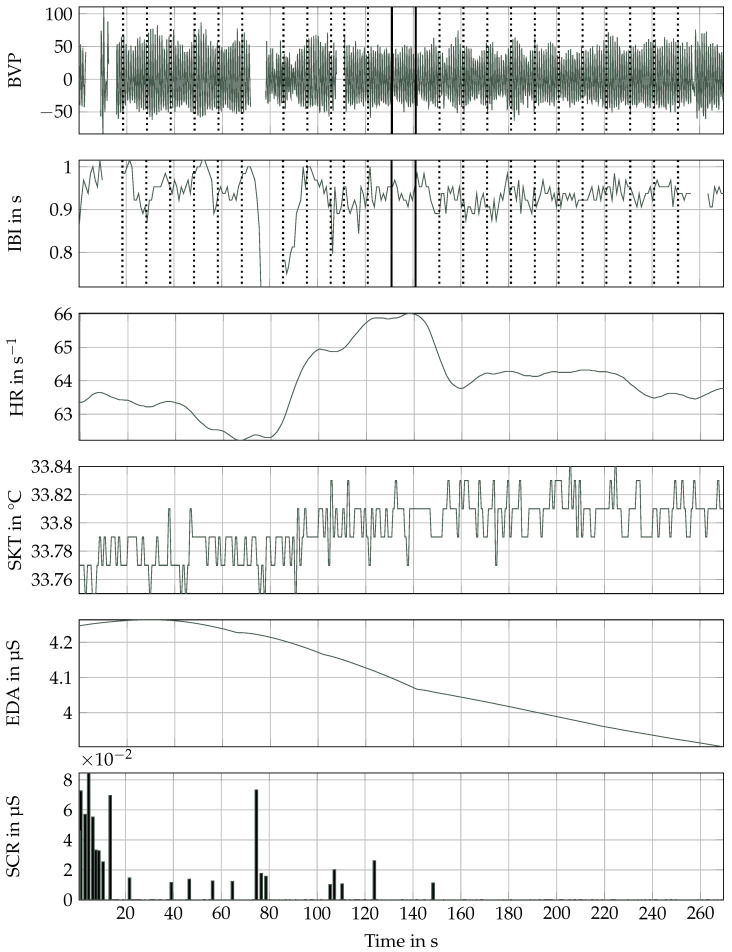
Overview of all preprocessed signals and segmentation. The placement of all viable segments with a length of 10 s is visualized for the inter-beat interval and blood volume pulse data with a dotted line, while the selected segment closest to the middle is marked with a solid line.

The EDA signal was processed using a 2nd-order Butterworth lowpass filter with a cutoff frequency of 1 Hz. We then extracted the skin conductance response (SCR) signal from the preprocessed EDA signal using the continious decomposition analysis (CDA) as described by Benedek and Kaernbach [30]. After the CDA, it is important to apply a threshold for the minimum amplitude of the detected peaks to avoid misinterpreting noise as SCRs [30]. While most commonly, a threshold of 0.05 μS is applied [30], we observed that numerous participants didn’t present many SCRs above 0.05 μS. As a result, we lowered the threshold to 0.01 μS, which remains within acceptable limits according to the Empatica recommendations [31]. The SKT signal was processed using a 2nd-order Butterworth lowpass filter with a cutoff frequency of 1 Hz. The resolution of the SKT signal and the accuracy of the sensor are 0.02 °C. Consequently, in Figure 5, the observable variations are limited to a subtle shift in the baseline temperature.

After individual signal processing, all signals were segmented. Given our goal of using feedback as input for a controller, immediate feedback is ideal. As such, we opted for shorter segments and selected a segment length of 20 s for the subject-independent approach, but also tested segment lengths between 10 s and 40 s. We utilized the freedom granted by the 300 s duration of each emotion segment to avoid the use of noisy data. Firstly, we removed the initial 30 s from each segment to allow for the time it might take subjects to immerse themselves in the situation and truly feel the corresponding emotion. Secondly, we identified all uncorrupted segments that meet the required length, noting that only the BVP and IBI signals were filtered for outliers. We then selected the segment closest to the midpoint of the remaining 270 s worth of signal.

The relatively lengthy duration of the emotion elicitation also offers the opportunity to extract multiple segments from each emotion segment, providing more training data for the classifier, as has been done in several studies. However, this approach requires caution as these segments won’t be interspersed with distraction tasks intended to reset the emotional baseline. To somewhat counteract this, we decided to consider only segments that are at least 10 s apart. Figure 5 displays all preprocessed signals, illustrating both taking a segment from the middle (indicated by a solid line) or multiple segments (represented by dashed lines) from the uncorrupted parts of the signal.

### 2.3. Feature Engineering

In this study, we decided to extract a broad set of features manually selected from related studies that worked with similar signals [5,7,11,12,18,27]. An overview of these features can be seen in Table A1. Only those features that appeared meaningful were selected. For instance, some features extracted from the SKT were suspected to be influenced by a baseline drift, and no frequency-domain features were extracted from the IBI signal, given they don’t provide meaningful information when computed for a short segment. Some features for the BVP signal were computed using Welch’s power spectral density (PSD), which is a noise-reducing method for spectral density estimation, implemented in Matlab [32].

As a filter method for features, we implemented the ReliefF algorithm in Matlab with ten nearest neighbors in the subject-independent approach. The ten features with the highest predictor importance weights were subsequently selected. We preferred to use a filter algorithm like ReliefF, which selects features, over projecting the data into a low-dimensional space, as the latter approach might fail to select meaningful features due to a possible non-linearity, or otherwise. Therefore, we used a principle component analysis (PCA) algorithm with a polynomial kernel as a reference using a toolbox [33]. We also utilized sequential forward selection (SFS) and sequential backward selection (SBS), implemented in Matlab. The algorithm iteratively selects a subset of features from the feature matrix by assessing a custom criterion. Typically, the loss of the classifier to be later trained on the reduced feature set is used, making wrapper methods classifier-dependent. To stabilize the results, we took the average of three cross-validations as the criterion.

Given that we used a distance-based classifier, scaling our features is advisable. To compensate for individual differences, we min-max-scaled each individual’s features, considering the maximum and minimum values of the extracted features across all emotions.

### 2.4. Classification and Validation

For classification and hyperparameter optimization, we implemented an SVM algorithm in Matlab. While using 10-fold validation is a very common approach, even when working with multi-subject data, for the purpose of generalizability one should perform LOSO validation to make sure that the model is not trained on data from the same subject as the test is performed on. Since we only extracted one segment per emotion per subject in the subject-dependent approach, we decided that *k*-fold validation was sufficient. However, for the multi-segment approach, we also tested our model using LOSO validation, where we manually split the data set into folds where segments from one participant were set as the test set and the rest of the segments were used as the training data set.

## 3. Results

In this section, we present the results from the preprocessing, feature engineering, and classification with different validation methods. We additionally split our results into the accuracy of the four emotions within the valance-arousal plane as well as the accuracy of the valance and arousal dimensions separately.

### 3.1. Preprocessing Results

This section presents the outcomes of our signal processing, focusing on two crucial aspects. Firstly, we chose not to interpolate the BVP and IBI data due to the potential presence of long sequences of missing data points, rendering interpolated data unreliable, which impacts the number of segments available for classification. Secondly, we encountered difficulties in detecting SCRs in the EDA data.

**Figure 6:**
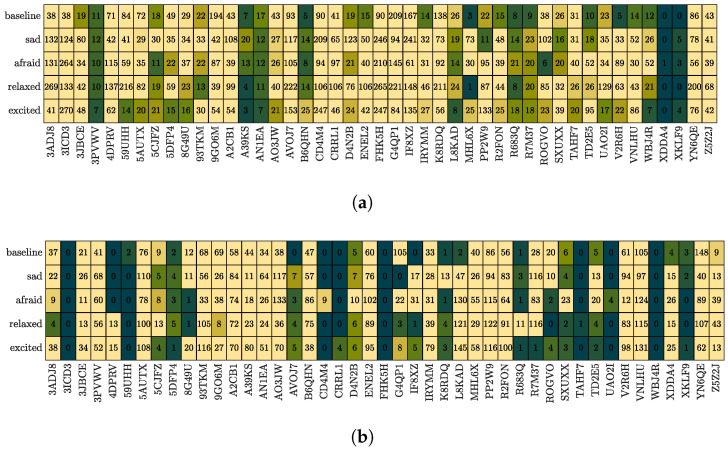
Corruption of multi-subject data set visualized as heatmaps. Darker colors indicate a signal that is noisy or unclear. (**a**) The longest segment length for all emotion tasks in seconds (the maximum value for the colorbar is set to 30). (**b**) The number of SCRs with an amplitude above 0.01 μS per emotion task (the maximum value for the colorbar is set to 10).

Figure 6 provides an overview of the corruption of the BVP. In Figure 6a, we visualize the longest uncorrupted segment for each emotion task and each participant. The color scale’s upper limit was set to 30 s. The data corruption appears to be subject-dependent, with some participants’ data almost entirely corrupted (e.g., XDDA4), while others are almost completely uncorrupted. Figure 6b presents the number of detected SCRs during each emotion task with an amplitude above 0.01 μS. The color scale’s upper limit was set to ten, revealing some participant data sets with very few SCRs (e.g., 3ICD3). This is in spite of our choice to use an unconservatively low threshold for detecting peaks, suggesting that we may risk identifying insignificant SCRs. Again, corruption does not seem to occur randomly and some sub-data sets appear more affected than others.

Given our approach of excluding data without an uncorrupted BVP segment of the required length, the size of the data set depends on the segment length. For a segment length of 20 s, which was used for subject-dependent testing, the data set consists of:

- 134 uncorrupted segments (out of 180 segments),
- Data from 40 participants (out of 45),
- Label distribution for emotions: Afraid: 35, Excited: 31, Relaxed: 34, Sad: 34,
- Label distribution for arousal according to the SAM: High: 73, Low: 61,
- Label distribution for valence according to the SAM: High: 75, Low: 59.

Even though the segment length was chosen relatively low in comparison to the length of one emotion task (270 s), the number of segments for testing was reduced significantly.

### 3.2. Feature Engineering and Classification Results

**Figure 7:**
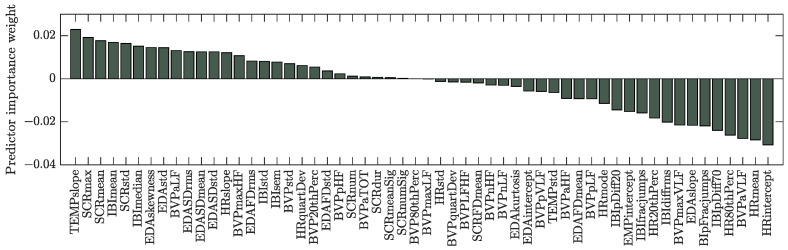
Predictor ranks computed with the ReliefF algorithm for the emotion label and segment length of 20s.

**Figure 8:**
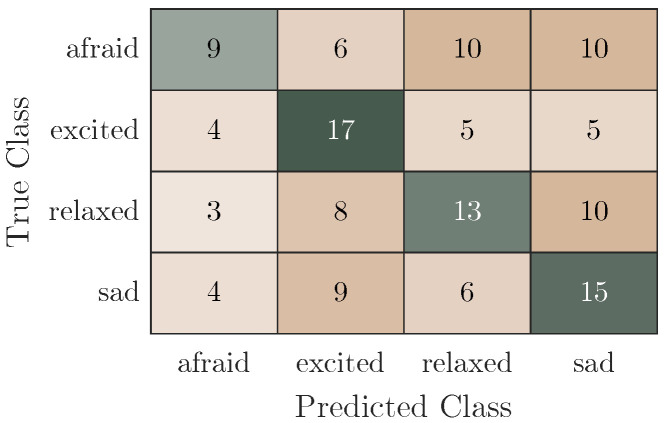
Confusion matrix for the classification for the emotion label, segment length of 20 s, 10-fold validation and a trained SVM with an accuracy of 39%. Green highlights the correctly classified samples and pink the incorrectly classified samples, where darker shades indicate a higher number of samples.

The predictor importance weights computed for the emotion label with the ReliefF algorithm can be found in Figure 7. Only the ten features with the highest weights were selected in the subject-independent approach. It is clear that selected features were extracted from all of the available sensors. Figure 8 shows the confusion matrices for all predictions from the 10-fold CV. While the classification for emotion performs better than random for all classes, the emotion “afraid” seems especially difficult to distinguish.

The results of the classifications for different data sets, feature sets, and validation methods are then displayed in Table 2. The accuracy of the trained model, defined as the average proportion of correctly classified instances averaged accross all test folds, is presented for classifications for the emotion labels, as well for binary classification (low/high) along the valence and arousal scales, according to the results of the self-assessments. Ten features were selected with the ReliefF algorithm from a segment of length 20 s as discussed above unless otherwise specified.

The first subdivision of the table shows classification results for segment lengths ranging from 10 s to 40 s. None of the trained models achieved accuracy significantly better than average guessing.

The second subdivision presents the outcomes for different feature selection methods: Feature selection with the ReliefF algorithm with five and fifteen features, and SFS and SBS. None of these methods significantly improved the results. Furthermore, it is essential to highlight that the use of SFS and SBS resulted in the selection or deselection of only a limited number of features, raising concerns about the robustness and meaningfulness of these feature sets. Given the marginal improvement in correct rates, it appears that the issue may not lie with the selected features.

The last subdivision contains the results of the multi-segment approach with segment lengths of 20 s and 10 s. The first data set (multi20) contains all segments with a minimum length of 20 s. The second data set (multi20 (5–10)) is similar, but the minimum and maximum numbers of extracted segments per emotion and participant were limited to a range of five to ten to minimize a bias toward participants with more uncorrupted segments. To maximize the number of segments, a third data set (multi10 (10–20)) was computed with a segment length of 10 s and a range of ten to twenty segments. Each of these data sets was tested using both 10-fold and LOSO classification.

The classifier’s accuracy did improve significantly, but only for the data sets with a limited range of segments. The best results were achieved for the multi10 (10–20) data set, with an accuracy of 75%. However, this was only the case if the model was validated using 10-fold validation, as the accuracy dropped to almost the same as random guessing with LOSO validation.

We see that the accuracy of our model improved notably when we extracted multiple segments from each emotion task. This improvement, however, was primarily observed when we constrained the range of segments for each emotion per participant and used *k*-fold validation. These findings suggest that the success of classification in emotion recognition might significantly depend on the similarity of the data between training and testing sets (e.g., segments with identical emotions and participants). This could potentially explain why the classifier performs better when there is a minimum number of segments to be extracted from each emotion task. In this way, the data set does not include segments from participants for which only a small amount of data is available.

## 4. Discussion

We can note how the subject-independent approach leads to an accuracy of our SVM classifier on segment lengths of 20 s that is just above random guessing both when considering four emotions (36%) and when considering only two of them (58% for one case and 62% for the other). Raising the rate of correct classifications requires taking a subject-dependent approach, i.e., using multiple segments from each emotion task per participant. In this case, using 10-fold cross validation, the correct rates increase to up to 75% for four emotions. This result can be then used for interpreting prior research, and highlight the issues that subject-dependency has in the previously published results in emotion recognition. The remainder of this section does thus such interpretation.

### 4.1. On Experimental Design

After preprocessing, a significant portion of the BVP and EDA data was found to be corrupted, reducing the usable data from 180 to 134 segments. While the problem of missing and noisy data due to movement has been reported in previous studies [7,20], the percentage is still surprisingly high considering that participants were asked to remain still. The lost data not only increased the time spent on the project but also potentially impacted our results. Despite SCR measures potentially providing valuable emotion indicators, only 126 segments had more than 10 SCRs, which could lower correct rates even if these measures were perfectly predictive. The overall amount of data corruption might be especially challenging for real-time applications. Another challenge is that writing about emotions is time-consuming, limiting the size of a single-session data set when using the AEMT.

### 4.2. Comparison to Previous Studies

Particularly with the machine learning methods, we purposely remained closely aligned with the literature, for instance, [6,7,8,10,11,12,24]. The accuracies achieved in these papers are claimed to be of 80% and above, see Table 1. However, it is challenging to make direct comparisons to other studies due to differences in signals used, emotion elicitation methods, and subject dependency across studies.

We identified two studies that used Empatica devices similar to ours. Gjoreski et al. [7] achieved around 73% accuracy for three stress levels and 90% for two stress levels. The study conducted by Li et al. [18] effectively identified periods of intense stress; however, since they used a regression model instead of a classification model, it is difficult to directly compare their results to our study. These studies demonstrate Empatica’s potential for stress measurement, but it’s unclear if these methodologies are transferable to emotion detection.

Chanel et al. [24] used a recall scheme similar to ours and achieved 50% accuracy for four classes and 70% for binary classifications compared to our best results of 75% and 85%, respectively. Their approach differed, as they used EEG data, trained a subject-dependent model, and collected more data due to shorter emotion tasks. The study by Picard et al. [13] also used a recall setup and achieved 81% accuracy with a k-nearest neighbours (kNN) classifier, but it only involved a single subject.

While these studies demonstrate the potential utility of the different components within our experimental design for emotion recognition using physiological signals, to the best of our knowledge, there has been no successful demonstration of subject-independent emotion recognition using a recall scheme. We take this a step further by asserting that many studies based on multi-subject datasets may create a misleading impression of favorable subject-independent results, while our findings emphasize the significant impact of subject-dependency on these outcomes. We will discuss this in detail below.

### 4.3. On Subject-Dependency

While the results for the multi-segment approach are encouraging, it is important to raise concerns regarding the fact that these segments originate from a single emotion task. Our observations, however, serve as evidence that emotion recognition is highly subject-dependent. Our assertion that subject dependency plays a critical role in emotion recognition gains further support upon examining Table 1. While nearly all researchers employ *k*-fold validation, the majority conduct multiple trials per emotion. As a result, the training data set contains information similar to that in the test data set. Notably, some studies even extract multiple segments from one emotion task [5,9]. Our analyses suggest that this practice disregards the importance of LOSO in obtaining subject-independent, and, hence, generalizable results.

By examining our data using both a subject-dependent and a subject-independent approach, we believe to have successfully demonstrated the strong subject dependency in emotion recognition. Results from literature with high accuracy and data sets collected from multiple subjects might thus provide too optimistic performances and achievable correct rates. This optimism is likely due to uncertainty regarding whether validation is consistently performed with data from an entirely new subject. Our study, however, clearly shows the need to collect subject-dependent data.

In addition to our best-performing model’s inherent subject-dependent nature; rendering it unsuitable for extrapolation to individuals who are not present in the dataset; there exists an element of uncertainty surrounding its temporal stability and its adaptability to diverse demographic cohorts. This concern is not unique to our model but extends across the field, hence requiring further investigation.

## 5. Conclusions

Our first main objective was to establish a minimally invasive setup to advance technology readiness levels in emotion recognition, particularly to provide emotional feedback during medical and non-medical treatments involving emotional and psycho-physiological feedback. The second main objective was to examine the impact of subject dependency by working with different methods for segmentation and validation.

Relatively to the first objective, the experience gained through the collection and processing of our data may be summarized in a set of experimental design recommendations. In primis, due to the sensors’ high sensitivity to noise, we advise against using the Empatica E4 device in environments with significant movement or where rapid emotion classification is essential, such as in control-oriented scenarios. For instance, from a total of 180 segments with a length of 270 s, we were only able to extract 134 uncorrupted segments with a length of 20 s. Moreover, our literature review suggests that issues like segmentation and sample size may have larger importance than what they seem to be typically credited with. We thus collected some advice on which data set sampling approaches one may follow in our discussion section.

Relatively to the second objective, we note that while the SVM we trained with a subject-independent data set barely performed better than random guessing (36% for four emotions). However, we achieved good results when we extracted multiple segments from each emotion task for each participant (75% for four emotions). This strategy obviously compromises generalizability, but is nonetheless useful to collect evidence that strongly suggests that emotion recognition is affected by a highly subject-dependent issue. This finding, in turn, highlights the importance of taking individual differences into account in studies of emotion recognition.

Expanding the dataset by including a more extensive and diverse pool of participants is one avenue for improving subject-independent results; in line with the need for larger datasets when employing deep learning techniques. Furthermore, the incorporation of a broader range of sensors may enhance the approach at the possible expense of reduced experimental flexibility. It is crucial to acknowledge that, despite their success in some instances, these strategies may not ensure favorable outcomes, given that they have predominantly been evaluated within subject-dependent validation frameworks.

Still relative to our second objective, and considering that getting subject-independent results in emotion recognition using physiological signals is still a challenge, we propose to start data collection campaigns by focusing first on a single subject. In scenarios involving individualized, long-term therapies, developing personalized emotion recognition models could be advantageous. From an experimental design point of view, we also emphasize the importance of collecting multiple emotion segments from each participant. We also note that we cannot exclude the possibility that time variance may also play a crucial role; likely models will need to be trained in an online fashion at the beginning of each session.

The most suitable model for a participant might be chosen based on easily collectible metadata, potentially offering a more personalized and accurate approach to emotion recognition.

## Figures and Tables

**Table 1:** Segmentation approaches from a selected number of studies.

Author	Number of Segments	Number of Subjects	Number of Labels	Trials per Label	Segments per Trial	Validation	Model	Accuracy
[11]	111	37	3	1	1	10-fold	SVM	97%
[26]	360	3	4 *	30	1	LOO	EMDC	70%
[5]	4800	20	2	40 **	6	CV	NN	82.1%
[12]	198	33	3	2	1	LOSO	SVM	83.8%
[27]	477	101	5	75–134 ***	1	LOO	RF	74%
[24]	3000	10	3	100	1	LOO	SVM	50%
[13]	160	1	8	20	1	LOO	kNN	81%
[6]	192	32	2	3	1	20-fold	SVM	90%
[16]	ca. 288	36	4	8 **	1	10-fold	NN	75%
[19]	176	4	8	44 **	1	LOSO	RF	62.1%
[9]	ca. 12,800	32	2	40 **	10	10-fold	CNN	87.3%

* four emotions were elicited for (binary) classification (valence/arousal); ** trials per subject for all labels; *** trials per label for all subjects; Abbreviations: LOO: leave-one-out, LOSO: leave-one-subject-out, CV: cross validation, SVM: support vector machine, EMDC: emotion-specific multilevel dichotomous classification, NN: neural network, RF: random forests, kNN: k-nearest neighbours, LR: logistic regression, CNN: convolutional neural network.

**Table 2:** Results for different data sets, feature sets and different methods of validations.

Name	Accuracy Emotions	Accuracy Valence	Accuracy Arousal	Validation	Segment Length	Number of Segments	Number of Features
seg10	35%	65%	52%	10-fold	10 s	161	10
seg20	39%	59%	63%	10-fold	20 s	134	10
seg30	37%	58%	49%	10-fold	30 s	106	10
seg40	37%	56%	59%	10-fold	40 s	86	10
ReliefF5	33%	53%	63%	10-fold	20 s	134	5
ReliefF15	26%	54%	57%	10-fold	20 s	134	15
PCA	27%	54%	56%	10-fold	20 s	134	10
SFS	37%	67%	65%	10-fold	20 s	134	3/1/4
SBS	38%	55%	49%	10-fold	20 s	134	55/52/53
multi20	44%	62%	60%	10-fold	20 s	484	10
multi20	23%	46%	53%	LOSO	20 s	484	10
multi20 (5–10)	57%	72%	80%	10-fold	20 s	276	10
multi20 (5–10)	24%	53%	58%	LOSO	20 s	276	10
multi10 (10–20)	75%	80%	85%	10-fold	10 s	597	10
multi10 (10–20)	32%	55%	52%	LOSO	10 s	597	10

## Data Availability

Data available on request due to privacy restrictions.

## Abbreviations

The following abbreviations are used in this manuscript:

ACC	acceleration
AEMT	autobigraphical emotion memory task
ANS	autonomic nervous system
BVP	blood volume pulse
CDA	continious decomposition analysis
CNN	convolutional neural network
CV	cross validation
DEAP	database for emotion analysis using physiological signals
DL	deep learning
ECG	electrocardiography
EDA	electrodermal activity
EEG	electroencephalography
EMDC	emotion-specific multilevel dichotomous classification
EMG	electromygraphy
EOG	electrooculography
HR	heart rate
IBI	inter-beat interval
kNN	k-nearest neighbours
LOO	leave-one-out
LOSO	leave-one-subject-out
LR	logistic regression
ML	machine learning
NN	neural network
PCA	principle component analysis
PPG	photoplethysmography
PSD	power spectral density
RESP	respiration rate
RF	random forests
SAM	self-assessment manikin
SBS	sequential backward selection
SCR	skin conductance response
SFS	sequential forward selection
SKT	skin temperature
SL	supervised learning
SVM	support vector machine

## Appendix A

**Table A1:** Overview of the 55 features selected for this study.

Name	Description	Name	Description
PPGmaxVLF	Highest power in VLF band	HRintercept	Intercept of a linear regression line
PPGmaxLF	Highest power in LF band	HR20thPerc	20th percentile
PPGmaxHF	Highest power in HF band	HR80thPerc	80th percentile
PPGaVLF	Raw area of VLF band	HRquartDev	Quartile deviation (75th–25th percentile)
PPGaLF	Raw area of LF band	EDAstd	Standard deviation
PPGaHF	Raw area HF band	EDAslope	Slope of a linear regression line
PPGaTOT	Total raw area of VLF, LF and HF bands	EDAintercept	Intercept of a linear regression line
PPGpVLF	Relative VLF area w.r.t. total area	EDAkurtosis	Kurtosis
PPGpLF	Relative LF area w.r.t. total area	EDAskewness	Skewness
PPGpHF	Relative HF area w.r.t. total area	EDAFDsqrtMean	Root mean square of first derivative GSR signal
PPGLFHF	Ratio of LF to HF power	EDAFDmean	Mean value of first derivative GSR signal.
PPGstd	Standard deviation	EDAFDstd	standard deviation
PPG20thPerc	20th percentile	EDASDsqrtMean	Root mean square of second derivative
PPG80thPerc	80th percentile	EDASDmean	Mean value of second derivative
PPGquartDev	Quartile deviation (75th–25th percentile)	EDASDstd	Standard deviation
IBImean	Mean value	SCRnum	Number of SCR
IBImedian	Median value	SCRmean	Average amplitude
IBIstd	Standard deviation	SCRnumSig	Number of significant responses ( 1.5 μS)
IBIsem	Standard error of the mean value	SCRmeanSig	Avergage amplitude of significant responses ( 1.5 μS)
IBIfracjumps	Number of successive RR differing by greater or equal to 50 ms	SCRdur	Average duration
IBIpFracjumps	Percentage of successive RR differing by greater or equal to 50 ms	SCRmax	Maximum amplitude
IBIdiffRms	Root mean square of successive RR differences	SCRstd	Standard deviation
IBIpDiff20/IBIpDiff50/IBIpDiff70	Percentage of the differences between adjacent RR samples that are greater than 20 ms/50 ms/70 ms	SCRFdmean	Mean of first derivative
HRmean	Mean value of heart rates	TEMPstd	Standard deviation
HRstd	Standard deviation heart rates	TEMPslope	Slope of a linear regression line
HRmode	Mode	TEMPintercept	Intercept of a linear regression line
HRslope	Slope of a linear regression line		
